# Quality Evaluation of Muffins Enriched With Blueberry and Cranberry Powders

**DOI:** 10.1155/2024/7045401

**Published:** 2024-08-12

**Authors:** Njomza Gashi, Endrit Hasani, Salih Salihu

**Affiliations:** ^1^ Department of Food Technology With Biotechnology Faculty of Agriculture and Veterinary University of Prishtina “Hasan Prishtina”, Prishtina, Kosovo 10000; ^2^ Faculty of Agricultural and Food Sciences and Environmental Management University of Debrecen H-4032, Debrecen, Hungary

**Keywords:** blueberry powder, color, cranberry powder, muffins, physicochemical parameters, polyphenols, sensory evaluation, texture

## Abstract

The use of plant extracts to enhance the nutritional profile and consumer appeal of food products has attracted considerable attention within the food industry. This is due to the fact that its high composition with bioactive ingredients affects the increase in nutritional value and the general acceptability of the product. The main focus of the food industry and researchers remains bakery products due to their high level of consumption and the suitability of combining them with different fruits. Given the prominence of bakery products in consumption and their compatibility with various fruits, investigating the enrichment of muffins with plant extracts is crucial. This was the main objective of this study, to improve the nutritional profile of muffins through berries' incorporation and evaluate the consumers' acceptability for these novel products. In this study, the muffins were enriched with cranberries and blueberries in powder form at concentrations of 3%, 6%, and 9%. Physicochemical parameters (such as weight, height, specific gravity, moisture, water activity, pH, and acidity), color, texture, and sensory parameters were analyzed in these samples, which were also compared with control samples without blueberry and cranberry powder. The results showed that pH and acidity were significantly different (*p* < 0.05) by the addition of blueberry powder, with pH values getting decreased and acidity values getting increased. The pH of the 9% enriched muffins measuring 5.38 and the acidity reaching 2.47 indicated a notable impact of extracts on the overall acidity level of the products showing comparable results to control samples. Hardness and gumminess as the main texture properties showed notable increase with the addition of blueberry and cranberry powders. Compared to control samples with 8.79 value of hardness, they achieved much higher values, 15.32 for cranberry and 10.58 for blueberry muffins, respectively. Similarly, they scored significantly higher values for gumminess, where cranberry muffins scored the value of 4.39 and blueberry muffins scored the value of 3.38, while control samples scored the value of 3.02. Furthermore, a direct relationship was observed between the concentration of these extracts and redness (*a*^∗^), while the ratio is inverse for the parameters of lightness (*L*^∗^), yellowness (*b*^∗^), chroma (*C*^∗^), and the value of hue angle (*h*). This factor is crucial to fulfill consumers' requests for more attractive attributes of such products not only in taste but also in their physical appearance. Therefore, even the sensory evaluation has shown that consumers like samples with higher amounts of cranberry and blueberry, respectively, with 6% (51.7% for blueberry muffins) and 9% (43.3% for cranberry muffins). The evaluation of blueberry samples revealed that the control sample was not the least preferred, primarily due to the lack of a well-defined taste in muffins with 3% extract; however, in the case of cranberry muffin evaluation, the control sample emerged as the least liked, indicating consumers' acceptance of the changes in muffin formulation. This shows the impact of enriching such bakeries with berries for empowering the consumers' attitudes and such product functionality in markets. Exploring the previously unaddressed area of enriching muffins with these berry powders, this research provides crucial information on the advantages, challenges, and nutritional enhancements associated with such formulations, filling a significant gap in the existing literature.

## 1. Introduction

Bakery products have a special place on the tables of different populations around the world. In general, bakery industry is experiencing significant growth globally. In particular, products like bread, biscuits, and muffins are among the most popular snacks for all age groups including children. These are fundamental food items that encompass nearly all the essential nutrients required for human sustenance and well-being, including proteins, complex carbohydrates, calcium, iron, phosphorus, and B vitamins such as thiamine, niacin, and riboflavin [1]. However, there is a rising demand for bakery items rich in dietary fiber and protein due to health concerns, as traditional products are mainly made from refined wheat flour and lack these nutritional components [2]. With the expanding production of bakery goods, there is a need for the industry to innovate and fortify bakery products to meet the preferences of health-conscious customers. Muffins, as a sweet baked product, are highly valued by consumers for their physicochemical characteristics [3]. They hold a significant position in the bakery industry, with their popularity increasing steadily, also because of their spongy texture [4]. The main ingredients in the preparation of muffins are flour, eggs, yogurt, oil, and other ingredients that can depend on the recipe of different regions.

In recent times, the preparation of these products has been increasingly done with the enrichment of various herbal ingredients, which, among other aspects, bring numerous health benefits. Various studies emphasize the utilization of anthocyanin-rich fruits as primary ingredients for creating enriched products like bread and tortilla, providing consumers with alternative options [5]. Herbal extracts are formulated with a diverse combination of elements, encompassing essential oils alongside other important constituents such as tannin substances, resins, enzymes, organic acids, pigments, vitamins, minerals, and beyond [6]. Studies have shown that herbal ingredients improve the general characteristics of baked products. According to Acun and Gül [7], the addition of grape pomace and grape seed flours to the cookie composition influenced the increase in the dietary fiber values, and, in addition to the impact on nutritional value, the positive impact was also observed on the sensory attributes of the product. In addition to the incorporation to increase the palatability and nutritional value of the products, plant extracts such as lemon, rosemary, and oregano have been shown to be effective in preservation due to their powerful antimicrobial and antioxidant activity [8]. Given the efficacy of these plant extracts, the use of blueberries and cranberries also holds immense promise in bakery applications, offering not only nutritional enrichment but also natural preservation benefits, thus aligning with the growing demand for healthier and longer-lasting bakery products.

Based on this, blueberries and cranberries have great potential to be used as important ingredients in bakeries. Typically, fresh blueberries are composed of 84% water, 9.7% carbohydrates, 0.6% proteins, and 0.4% fat, and just in 100-g fresh blueberries, the average energy value is 192 kJ [9]. Berries, such as blueberries, are an important source of polyphenols. They are especially rich in anthocyanins [10]. Recent research indicates that eating blueberries for their anthocyanins reduces biomarkers and the risk of diseases, including cardiovascular disease, Type 2 diabetes, and cognitive decline [11]. Blueberries' anthocyanins have the ability to affect antioxidation, anti-inflammation, and insulin sensitization [12]. Blueberries are rich in lutein and vitamin C which are important dietetic components for nervous system health [13]. These components found in blueberries exhibit anti-inflammatory and vasoprotective properties, which help maintain balanced glucose delivery to insulin-sensitive tissues and promote healthy metabolic function. These aspects have implications in multiple areas of healthy aging [11]. According to a study by Guijarro-Fuertes et al. [5], the incorporation of blueberries' pulp into bread formulation resulted in variations in physicochemical characteristics, including changes in texture and antioxidant capacity, with the gluten network potentially playing a protective role for antioxidant compounds contributed by the blueberries. Additionally, blueberries provide a significant amount of dietary fiber, about 3%–3.5% of their total weight [9].

Furthermore, cranberries play an important role in the regulation of energy homeostasis, glucose and lipid metabolism, and systemic inflammation, and these affect the maintenance of cardiovascular health [14]. Cranberries have a rich chemical composition of phytochemicals, making them an excellent choice for inclusion in bakery products to enhance their nutritional profile and promote health benefits [15]. The value of this fruit increases even more due to its chemical composition, also because it has an effect in preventing urinary tract infections and maintaining gastroenteric and heart health [16]. Cranberries are rich in numerous bioactive compounds with antioxidant properties. They contain a wealth of phytochemicals, including phenolic acids, anthocyanins, flavones, flavonoids, and organic acids. Notably, cranberries are one of the few fruits high in proanthocyanidins, which prevent *Escherichia coli* from adhering to the urinary tract. Consuming cranberries can help prevent tooth decay, lower inflammation in the body, maintain healthy digestion, and lower cholesterol levels [17]. Cranberry fruits contain significant levels of triterpenoids, notably ursolic acid and oleanolic acid, which are concentrated in the fruit's skin wax and contribute to its anti-inflammatory, antitumor, and anticancer properties. The concentration of these triterpenoids varies among cranberry cultivars, but overall, their levels increase as the fruit matures, with ursolic acid content ranging from 22.7% to 32.2% of total triterpenoids [17].

All of these effects on the final food product and consumer health make the production of foods enriched with blueberry and cranberry powders increasingly important. Incorporating blueberry and cranberry powders into muffin formulations offers a multifaceted approach to improve overall quality of the product. The rich dietary fiber content of blueberries and cranberries can positively influence the textural properties of muffins, imparting a desirable moistness and crumb structure while also improving their overall nutritional profile. Furthermore, the high content of polyphenols in these berries not only contributes to their antioxidant properties but also introduces a variety of potential benefits to the muffins themselves. For example, the antioxidant activity of blueberry anthocyanins and cranberry phytochemicals could play a crucial role in extending the shelf life of muffins by mitigating the oxidative deterioration of fats and oils within the batter [18]. Furthermore, according to Wang et al. [19] tannins, which are naturally occurring polyphenols, positively affect dough mixing characteristics. This is achieved by encouraging noncovalent bonds among gluten proteins and initiating structure changes that favor the development of *β*-turn and *α*-helix formations while diminishing *β*-sheet configuration. These alterations enhance the texture of the dough and refine the microstructure of gluten networks, indicating the potential utility of tannins as an innovative enhancer of flour quality [19]. Additionally, the unique sweet-tart flavor profile characteristic of these berries can impart a delightful sensory experience to the muffins, appealing to a wider consumer base and elevating their appeal to the market. Another class of polyphenols are anthocyanins, which are highly present in blueberries and cranberries. Anthocyanins not only are responsible for the vibrant color of berries but also act as natural pigments. When incorporated into muffins, these polyphenols can help retain the natural color of the berries, imparting an attractive visual appeal to the final product. Therefore, the objective of our study was the development of muffin formulations enriched with blueberry and cranberry powders. These berries were chosen as key ingredients due to their nutritional richness, particularly their high dietary fiber content and elevated levels of polyphenols, which contribute to significant antioxidant properties. By incorporating these powders into muffins, we aimed to enhance the nutritional profile and infuse the product with the distinct sweet-tart flavor note characteristic of these fruits [20]. Samples of the enriched muffins were analyzed for various physicochemical properties, including moisture, dry matter, weight, height, specific gravity, water activity, and acidity. Additionally, the polyphenolic content was measured to evaluate the enrichment effect of the blueberry and cranberry powders. The sensory evaluation focused on taste, odor, color, mouthfeel, and external appearance, while the texture analysis included measurements of hardness, cohesiveness, and gumminess. The popularity and positive consumer perception of blueberries and cranberries make them attractive choices, enhancing market appeal and perceived health benefits of the muffins. By selecting these ingredients, we aim to improve both the nutritional value and sensory experience of the final product. Through sensory evaluation, we sought to assess consumer preferences regarding the incorporation of these blueberry and cranberry powders into muffins, aiming to gauge market acceptance and the potential for product commercialization. Furthermore, our study quantified the polyphenol content in muffins enriched with blueberry and cranberry powders to elucidate the potential health benefits associated with these antioxidant-rich ingredients. The effect of these powders on the sensory properties (taste, odor, color, mouthfeel, and external appearance) and textural properties (hardness, cohesiveness, and gumminess) of the muffins was thoroughly investigated. All samples were analyzed for their physicochemical properties, texture, and polyphenol content. Successful incorporation of such berries into muffin formulations shows a promising approach to enhance the nutritional profile of baked goods, which could significantly influence future product development in the food industry. This suggests that manufacturers can create healthier, more appealing products that cater to consumer demands for nutritious and flavorful foods. This could also encourage industry practices to prioritize the use of natural ingredients with high nutritional profile, potentially leading to a broader adoption of these formulations.

## 2. Materials and Methods

### 2.1. Muffin Preparation

**Figure 1:**
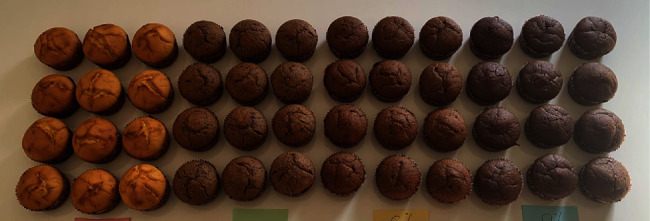
Muffin samples: control, with 3%, 6%, and 9% blueberry powder.

The research is based on the comparison of the attributes of the control muffin samples (without blueberry and cranberry powder) and those with 3%, 6%, and 9% blueberry and cranberry powder. The control muffins were made following a standardized recipe, featuring a blend of essential ingredients: white flour (25%), yogurt (33.3%), eggs (4.4%), oil (8.3%), baking powder (1%), sugar (16.7%), and vanilla extract (0.8%). All ingredients were thoroughly mixed for 5 min until a homogeneous batter formed. Subsequently, the batter was evenly portioned into 12 muffin tins, maintaining a consistent size and distribution between batches (89 g each). The recipe for the control muffins and enriched muffins is detailed in Table 1, which outlines the proportions of each ingredient used in the preparation process. All these ingredients were mixed until a homogeneous mass formed and then baked at 180°C for 30 min, ensuring uniform heat distribution and thorough baking of the muffins. They were baked using a Kolb brand convection oven model with outside dimensions of 600 × 720 × 558 mm. In this convection oven, the mode of heat transfer involved the circulation of hot air throughout the oven cavity. This airflow facilitated the even heating and cooking of the muffins, ensuring consistent results on all trays and levels. Meanwhile, for the preparation of the samples with 3%, 6%, and 9%, the same recipe as defined in Table 1 was applied, with the only modification being the replacement of white flour with blueberry and cranberry powders (Figure 1). The replacement of white flour with blueberry and cranberry powder was conducted by weight, and the overall weight of the muffin mixture remained consistent across all variations, ensuring uniformity in muffin sizes.

When selecting concentrations of 3%, 6%, and 9% for blueberry and cranberry powders, our aim was to represent a range of enrichment levels that would allow a meaningful evaluation of the impact on muffin quality. These concentrations were chosen based on previous studies in the field, suggesting that lower concentrations may not provide sufficient flavor or nutritional enhancement, while higher concentrations could potentially lead to undesirable changes in texture or taste [21]. Additionally, considering the solubility and dispersibility of the powders, we aimed to ensure that the chosen concentrations would effectively incorporate into the muffin batter without compromising the overall quality of the product. This rationale guided the selection process to establish a balance between achieving significant enrichment and maintaining desirable sensory and textural properties in the final muffin products.

Blueberry and cranberry powders, obtained from Arctic Flavors in Espoo, Finland [22], and Tallinn, Estonia, respectively [23], were utilized in the muffin formulations, with detailed nutritional information provided in Table 2.

### 2.2. Estimation of Physicochemical Properties

Samples of muffins with blueberry and cranberry powder were analyzed for moisture, dry matter, and their weight and height. The specific gravity of the muffins was measured using a pycnometer, which was measured empty and then filled with muffin crushed sample and finally with water. The final specific gravity was determined using the following formula:

 $$\begin{array}{c} D=\frac{S-b}{a-b}\times 100 \end{array}$$

where *D* is the specific gravity, *S* is the pycnometer weight with sample, *a* is the pycnometer weight with water, and *b* is the pycnometer weight [24].

Another parameter examined was moisture, which was assessed using the loss on drying method, involving the application of a constant temperature of 130°C until the weight stabilized [25]. The water activity of the samples was measured using the Novasina LabMaster-aw neo-type instrument, while the acidity was measured using the titration method with 0.1 normal NaOH solution. Three grams of sample was mixed with 27 mL of distilled water [26], and phenolphthalein was used as an indicator. The acidity levels of the muffin samples were assessed through a process that involved the use of a pH meter. This involved dissolving 10 g of sample in 10 mL of distilled water, following which pH measurements were taken and recorded. In addition, total polyphenols were quantified using the Folin-Ciocalteu reagent as the primary chemical agent for the determination [27]. All measurements were carried out in triplicate.

### 2.3. Color Measurement and Texture Properties

Color attributes such as redness (*a*^∗^), lightness (*L*^∗^), and yellowness (*b*^∗^) were measured using a CR-410-type colorimeter. Each sample was subjected to five measurements, and from these readings, the chroma value (*C*^∗^) and the hue angle (*h*^∗^) were calculated using the provided equations.

$$\begin{array}{cc} \; & \text{Chroma}:C^{*}={\left[{\left(a^{*}\right)}^{2}+{\left(b^{*}\right)}^{2}\right]}^{1/2}\\ \text{Hue angle}:h^{*}=\tan^{-1}\left(\frac{b^{*}}{a^{*}}\right) \end{array}$$

Measurements such as chroma and hue angle are essential in this study as they provide valuable insights into the color attributes of the product, which play a crucial role in determining consumer preferences and overall sensory experience. In particular, chroma value indicated the purity of a color, while hue angle shows differences in color calculating redness and yellowness. Calculating the total color difference (*E*) between the groups involved using the values obtained for lightness (*L*^∗^), redness (*a*^∗^), and yellowness (*b*^∗^), following the procedure outlined by Knispel [28] and employing the equation provided.

 $$\begin{array}{c} \Delta E=\sqrt{{\left(L^{*}-{L_{0}}^{*}\right)}^{2}+{\left(a^{*}-{a_{0}}^{*}\right)}^{2}+{\left(b^{*}-{b_{0}}^{*}\right)}^{2}} \end{array}$$

Texture measurements such as hardness, cohesiveness, and gumminess were made using a TA.XT Plus texture analyzer (Stable Micro System, Surrey, United Kingdom) applying Texture Expert software for Windows (Stable Micro System) for processing data.

### 2.4. Sensory Evaluation

Sensory parameters were also analyzed through organoleptic tests, in which the hedonic scale was used to evaluate the taste, odor, color, mouthfeel, and external appearance of muffins. A group of 60 individuals, not trained in sensory evaluation, hailing from the University of Prishtina's Department of Food Technology with Biotechnology, was requested to provide their ratings on a 9-point hedonic scale. This scale ranged from 1, indicating *extreme dislike*, to 9, representing *utmost liking*, for all parameters under consideration. The decision to include nontrained panelists in our sensory evaluation was deliberate, aimed at involving students specializing in food technology who possess a basic understanding of sensory evaluation techniques. We believed that their familiarity with the subject matter would lead to more insightful and reliable assessments, mimicking real-world consumer responses. Prior to the evaluation, participants underwent a brief training session where they were introduced to the sensory evaluation process, including its components such as taste, odor, color, mouthfeel, and external appearance. They were provided with guidelines on how to use the hedonic scale and were instructed on maintaining neutrality throughout the evaluation process. This preevaluation training was essential to ensure consistency and reliability in their assessments, thereby enhancing the validity of the sensory data obtained. Of the sensory evaluators, 71.67% were women and 28.33% were men, all participants falling within the age range of 18–25 years old. The samples were ordered at room temperature and coded with three-digit codes. It should be noted that the order of presenting samples was mixed, rather than following the conventional sequence of control, 3%, 6%, and 9% variations. This deviation from the typical presentation order was implemented to prevent any bias that might arise from the panelists knowing the exact composition of each sample beforehand. During the sensory evaluation process, the palates of the panelists were neutralized with salt crackers and water.

### 2.5. Statistical Data Analysis

Data collected from the experiment were analyzed using IBM SPSS (Version 26.0, Armonk, New York, United States, 2020). The data was analyzed through both analysis of variance (ANOVA) and the general linear model (GLM). To test the normality of residuals, the Kolmogorov–Smirnov test (*p* > 0.05) was applied (*p* > 0.05), while Leven's test was used to check the homogeneity of variances (*p* > 0.05). Depending on whether the homogeneity of variance was violated or not, the Tukey post hoc test or the Games–Howell test was applied. If the assumption of homogeneity of variances was met (*p* > 0.05), the Tukey post hoc test was applied. However, if the assumption of homogeneity of variances was violated (p <0.05), indicating unequal variances between groups, the Games–Howell test was used. These statistical tests were applied for both physicochemical and sensory parameters.

## 3. Results and Discussion

### 3.1. Physicochemical Analysis of Muffins

The results for the physicochemical parameters of the muffins are presented in Table 3. Based on the results obtained, significant differences in the specific gravity of the samples were observed (*p* < 0.05). Specifically, samples containing 9% blueberry and those with 3%, 6%, and 9% cranberry exhibited higher values compared to control samples. Similarly, Nath et al. [29] stated an increase in the specific gravity of the samples compared to the control sample in the case of adding red capsicum pomace powder to muffins. Blueberry and cranberry powders have different densities compared to other ingredients such as flour, sugar, and eggs. When these powders were added to the batter, they altered the overall density of the mixture, leading to changes in specific gravity. Blueberry and cranberry powders when added to muffins may have introduced additional moisture, and this made the batter denser, potentially increasing the specific gravity. Changes in specific gravity can affect the distribution of air bubbles within the batter, which can lead to variations in the crumb structure and overall mouthfeel of the muffins. This can potentially affect consumer perception of the quality of muffins, as a denser or lighter texture may be preferred depending on individual preferences. Height measurements did not reveal substantial differences between samples; however, samples containing 6% and 9% cranberry stood out with higher values compared to the control sample. Furthermore, for moisture, the samples with more blueberry and cranberry powders, respectively, the samples with 6% blueberry, 3% cranberry, 6% cranberry, and 9% cranberry have marked significantly higher values than the control. This may be due to the high water content that the berries have, like most other fruits. Especially for moisture, another research on muffins with avocado puree as a fat replacer has also reported that it can be directly affected by the addition of blueberry and cranberry powders because of the high water content [30]. These berries have high levels of sugars which usually act as humectants [31]. This can influence the higher interaction with water inside the muffin's matrix. The higher moisture content of these fruits can lead to changes in the texture of the muffins, affecting their overall quality. Increased moisture levels may result in a softer crumb texture, potentially improving the perceived freshness and moistness of the muffins. However, excessive moisture can also contribute to a shorter shelf life by promoting microbial growth and accelerating staling processes. Therefore, careful control of moisture levels is crucial to maintain the desired texture and extend the shelf life of muffins.

The acids present in blueberries and cranberries can lower the pH of the muffin batter, which can affect various chemical reactions during baking and affect the texture and flavor of the final product. As far as pH and acidity are concerned, almost all samples showed significant differences from samples without blueberry and cranberry powders (*p* < 0.05). In the case of pH, all samples showed differences, while in the case of acidity, samples with 3% and 9% blueberry and those with 6% and 9% cranberry were distinguished. High acidity values were achieved under the influence of many acids that these extracts contain. Malic and quinic acids but especially citric acid are the main acids that are found in berries, being responsible for the most changes in pH and acidity in baked goods like muffins [32]. Also, sugars in muffins enriched with blueberries and cranberries can contribute to higher acidity during baking as they undergo reactions at high temperatures, releasing acidic substances. Nath et al. [29] also reported increased acidity in fortified muffins with red capsicum pomace powder from 2% to 10% extract. Additionally, in our previous study involving biscuits enriched with blueberry and cranberry powder, notable distinctions were observed. Specifically, biscuit samples containing 6% and 9% cranberry powder exhibited significant differences compared to control samples devoid of extracts [33]. Acidity levels can influence the overall sensory perception of the muffins, contributing to their tartness and flavor profile. However, excessive acidity may also lead to undesirable sensory attributes or reactions with other ingredients in the batter. On the contrary, no significant differences (*p* > 0.05) were observed between the samples in terms of the weight and water activity parameters.

### 3.2. Color Measurements

Color is an important parameter that greatly affects the evaluation of a food product. Tables 4 and 5 show the results of the color analysis of the crust and muffin crumbs enriched with blueberry and cranberry powders. Regarding the crust, the lightness decreased significantly (*p* < 0.05) the more blueberry and cranberry powders were added. In contrast, for redness, the results have increased significantly with the addition of extracts. There were no differences between the samples with 6% and 9% blueberries and cranberries. For yellowness, the result decreased significantly from 0% to 6% added extract (*p* < 0.05), while there were no differences between the 6% and 9% samples. Regarding the values of the chroma and *h* values, a significant difference was observed where the control samples had significantly higher values than the muffin samples enriched with blueberries and cranberries.

The comparison between blueberry and cranberry samples showed that there were significant differences (*p* < 0.05) in all color parameters. For the lightness yellowness and chroma values, the samples with blueberry showed significantly lower values than the samples with cranberry. For the redness parameters, the samples with cranberry showed significantly lower values than the samples with blueberry. For the *h* value, only the samples with 3% blueberry and cranberry powders showed differences, while the samples with 6% and 9% did not show significant differences.

On the contrary, significant differences were observed between the samples for the crumb characteristics (Table 5), depending on the percentage of blueberry and cranberry powders added. Thus, lightness and yellowness have decreased with increasing amount of blueberry and cranberry powders. Lightness and yellowness were higher in the control samples, and samples with 6% and 9% blueberry extract showed the lowest values. This is influenced by the content of the berries and also by the baking process. On the other hand, the redness parameter showed the opposite result, where the more the amount of blueberry and cranberry powders increased, its value also increased, registering significant differences between samples (*p* < 0.05). The chroma and *h* value parameters have registered a significant decrease of 0%–9% with the addition of blueberry powder to the muffin composition. Reißner et al. [34] also reported a decrease in lightness and chroma values in bread samples with berry pomace. Only in muffins with cranberry powder, no significant differences were recorded between the samples analyzed. The main components influencing these results are anthocyanins which significantly influence the color metrics. This is due to their strong pigmentation properties, contributing vibrant colors ranging from red to blue. Anthocyanins absorb the green and yellow light wavelengths (500–600 nm), and they reflect certain wavelengths of light, especially within the red and blue spectrum [35]. When these wavelengths are reflected, they combine with the absorption of green and yellow light by anthocyanins. This gives us the perception of purple color which is the most attractive attribute of muffins.

From the perspective of the two groups of muffins with cranberries and those with blueberries, significant differences were observed for all color measurements (*p* < 0.05). For lightness, yellowness, chroma, and *h* value, the muffin samples with cranberries have marked higher values compared to the muffin samples with blueberries, which were distinguished by higher values only for the redness parameter.

To show a clearer view of the changes in color parameters, the total change in color (*E*) between samples with different percentages of blueberry and cranberry powders was also analyzed (Table 6). According to the results achieved, the differences between the samples with 6% and 9% cranberry can only be seen by an experienced observer, while the total color differences between the other samples are of Category V, which means that the observer can distinguish two distinct colors. These results apply to the crust and crumbs of cranberry muffins.

As for blueberry muffin samples, the observer can distinguish two distinct colors when comparing the control sample with other samples enriched with different percentages of blueberries, while in the comparison between the sample with 6% blueberry and the one with 9% blueberry, a clear color difference is perceived.

These changes observed in muffins can be attributed to various factors associated with the addition of blueberry and cranberry powders. In particular, these berries contain natural pigments such as anthocyanins, which impart vibrant colors that range from red to purple. When incorporated into muffins, these pigments interacted with other ingredients and caused chemical reactions during baking, resulting in alterations in color attributes such as lightness, redness, and yellowness.

### 3.3. Texture Properties

**Figure 2:**
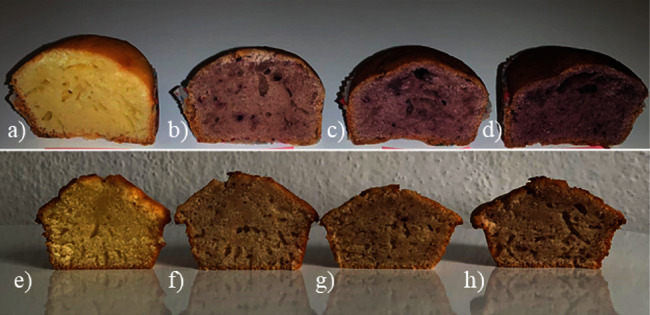
Muffin samples with different percentages of blueberry powder (BP) and cranberry powder (CB): (a) control, (b) 3% BP, (c) 6% BP, (d) 9% BP, (e) control, (f) 3% CP, (g) 6% CP, and (h) 9% CP.

Texture is another parameter that is evaluated in the selection of a product for consumption. For hardness, all cranberry samples showed differences from the main control sample, where the values showed a continuous increase but did not show significant differences among themselves. An increase in hardness was also reported by Grigelmo-Miguel, Carreras-Boladeras, and Martín-Belloso [36] who studied muffins with peach dietary fiber. In the same way, for the gumminess parameter, the samples showed differences from the control sample, which recorded the lowest value but did not show differences among themselves. Meanwhile, for coherence, only the sample with 9% cranberries differed significantly from the control sample (*p* < 0.05), while blueberry samples did not show significant differences (*p* > 0.05) for any of the texture parameters (Figure 2).

In the comparison between the two groups with different extracts, as can be seen in Table 7, significant differences have appeared for some of the parameters (*p* < 0.05). Therefore, for hardness and gumminess, cranberry muffins have shown higher results. Meanwhile, in terms of cohesiveness, significant differences were recorded between samples with 6% and 9% blueberry and cranberry powders. The presence of blueberry and cranberry powders in the muffin batter altered the distribution of the ingredients, affecting the overall structure and density of the muffins. The moisture content of the berries could also play a significant role, as it interacts with other ingredients during baking, influencing the hydration of starches and proteins and ultimately affecting the texture of the crumb. Furthermore, the acidic components of blueberries and cranberries react with baking powder, influencing the rise and texture of the muffins. Additionally, dietary fibers in blueberry and cranberry powders may have affected on the texture properties of muffins [37]. For instance, cellulose can increase the hardness of the final product, while pectin decreases the hardness with increasing viscosity. The combination of all these factors led to changes in the texture of the muffins, making them denser, moister, or crumblier depending on the specific characteristics of the berries and their interaction with other ingredients in the batter.

### 3.4. Total Polyphenol Content

Phenolic compounds are found in different amounts depending on the fruit analyzed. Fresh fruits have a higher content of phenolic compounds, as thermal processing reduces the amount of these chemical compounds [38]. This phenomenon is verified by the results of this research, where the muffin samples had lower polyphenol values. Polyphenol content has been observed to show significant differences from the powdered form of blueberries and cranberries to those included in muffin samples (*p* < 0.05) (Table 8). Fresh blueberry and cranberry powder samples showed a higher content of polyphenols, and more blueberry and cranberry powder was added; muffins showed a higher content of polyphenols, although without significant differences between them (*p* > 0.05). Similar polyphenolic values have also been found in studies of other berries as well [39]. Only the sample with 9% blueberry was found to have no differences with the blueberry powder sample and marked the highest polyphenol value among all groups. Even in the comparison of the results between the groups, it was observed that the muffins enriched with blueberry extract had higher polyphenol values than the cranberry samples. This is related to the higher content of polyphenols in blueberries, although the values are close to those of cranberries but also vary depending on the cultivar.

### 3.5. Sensory Analysis

**Figure 3:**
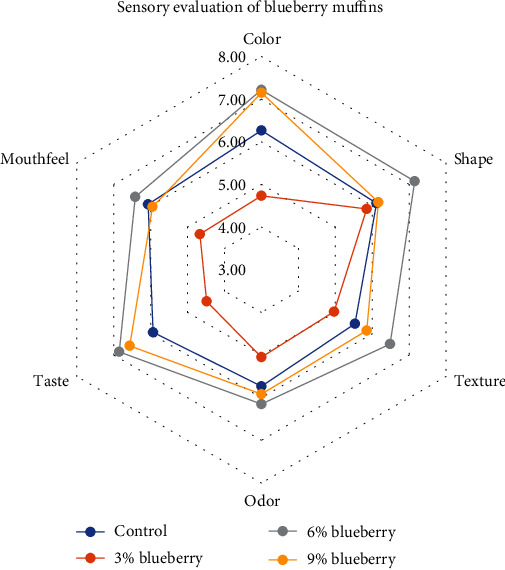
Sensory evaluation of muffins enriched with blueberry powder.

Extracts from plants are essential ingredients to improve the sensory attributes of bakeries [40]. In our study, sensory evaluators have shown different preferences for samples depending on the amount of blueberry and cranberry powders added. The more blueberry and cranberry powders, the more the product is liked by consumers in terms of taste, color, and shape. Significant differences in color evaluation were observed, where the evaluators liked the sample with 6% and the one with 9% blueberry more (*p* < 0.05). Higher preferences for enriched muffins may be associated with their perceived freshness. Vibrant colors, influenced by blueberry and cranberry powders, make the muffins more visually appealing, which can lead to the perception of better taste. This aligns with the physical measurements noted earlier, where the addition of berries increased the redness values of the muffins. Regarding the shape, differences appeared only in the sample with 6% blueberry, which is apparently more liked. Muffins with more appealing shapes are visually attractive, creating a positive first impression. Consumers often associate well-formed, visually appealing muffins with higher quality. Similar findings were observed in the evaluation of texture and taste, with no significant differences observed between samples containing 6% and 9% blueberries (*p* > 0.05) in these aspects. For other parameters such as aroma and mouthfeel, control samples and those with 6% and 9% blueberries did not show significant differences between themselves and the sample with 3% blueberries received the lowest score (Figure 3). In general, the most liked sample was muffin with 6% and blueberries with 51.7%, while the least liked sample was muffin with 3% and blueberries with 1.7%. In general, muffins with higher concentrations of blueberry powder (6% and 9%) were preferred in terms of color, probably due to the deeper and more intense coloration signaling richer flavor and higher nutritional content to consumers. Additionally, the texture of the muffins, influenced by the moisture content and the structure of the crumb, was optimally balanced in samples with 6% blueberry powder, contributing to higher satisfaction scores. Although taste and aroma did not differ significantly between muffins with 6% and 9% blueberries, overall liking scores were higher for these samples compared to those with lower concentrations (3%), suggesting a threshold of fruit powder concentration necessary for desired flavor intensity without overpowering the base flavor of the muffins.

**Figure 4:**
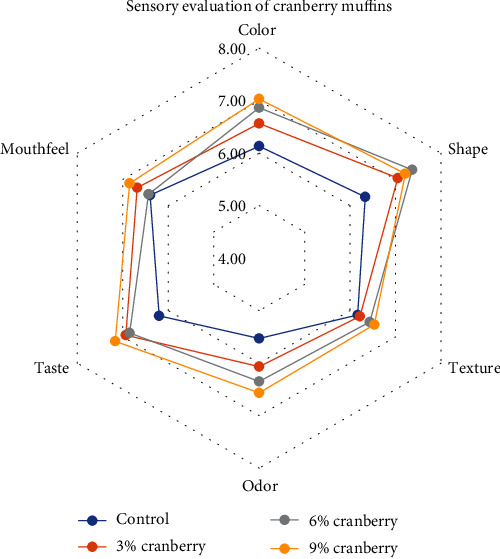
Sensory evaluation of muffins enriched with cranberry powder.

Even in the evaluation of cranberry muffin samples, a high evaluation was achieved for almost all samples for sensory parameters. Regarding color, the sample with 3% cranberry scored the lowest result in a significant way (*p* < 0.05), while the sample with 6% cranberry scored the highest score even though there were no significant differences with other samples (Figure 4). Regarding the shape of the sample with 6% cranberry, it received the highest evaluation and the one with significant differences from the control sample and the one with 3% extract, where the sample enriched with 3% was evaluated as the weakest for this parameter. This may be related also with the moisture content of enriched muffins. These muffins with 6% berries resulted with a slightly higher moisture content therefore tending to have a softer texture and richer flavor, enhancing the overall eating experience. This leads to higher satisfaction and preference. For texture, aroma, and taste, significant differences were observed in the evaluation between the control and 3% samples and those with 6% and 9% extract (*p* < 0.05). Muffins with 9% cranberry are rated with the highest points for texture, while those with 6% cranberry for aroma and taste. Similarly, Białek et al. [41] reported the high acceptability of muffins with pumpkin seed flour compared to control samples. Mouthfeel is another parameter included in the evaluation, where the sample with 9% cranberry received the highest rating, although there were no differences with the sample with 6% cranberry. On the contrary, both the control samples and those with 3% cranberry received the lowest scores, with no significant differences observed between them (*p* > 0.05).

The overall liking was greater for the 9% cranberry muffin samples with 43.3%, while the least liked sample was the control sample with 11.7%. These findings underscore the importance of cranberry powder concentration in improving various sensory attributes of muffins, ultimately influencing consumer preference and overall liking.

The findings of this study have significant implications both for the food industry and consumer health. The determination of optimal concentrations of blueberry and cranberry powders in muffin formulations not only enhances sensory characteristics but also improves the nutritional profile of the product. With higher concentrations of these powders positively impacting color, texture, odor, and taste, muffins fortified in this manner offer consumers a more appealing and flavorful product. Furthermore, the presence of antioxidants and other bioactive compounds in blueberries and cranberries suggests potential health benefits associated with their consumption. These muffins could serve as convenient vehicles to deliver fruit-based nutrients, contribute to a balanced diet, and potentially mitigate certain health risks. However, while the study highlights opportunities to develop functional and nutritious baked goods, challenges remain in scaling up production and commercializing these products. Factors such as cost-effectiveness, shelf life maintenance, market demand, and consumer acceptance pose significant considerations in the successful integration of these enhanced muffins into the market. Addressing these challenges and capitalizing on the opportunities presented by fortification of fruit powders could pave the way for innovative and nutritious bakery products that address evolving consumer preferences and health-conscious markets. The preference for higher concentrations of blueberries and cranberries in muffins suggests a strong consumer appeal for enhanced flavor and nutritional richness. This information could be leveraged in marketing strategies by highlighting the improved taste and potential health benefits of muffins enriched with higher blueberry and cranberry concentrations. Consumer education efforts could emphasize how these muffins offer a delicious way to incorporate important nutrients into their diet, promoting improved cardiovascular health, reduced inflammation, and enhanced antioxidant defense mechanisms. This is possible also through small labels in the muffins packed for markets. Findings of this study offer valuable insights for bakery product development, particularly in the formulation of commercial muffins. By determining the optimal concentrations of blueberries and cranberries, manufacturers can enhance both the sensory and nutritional values of their muffins. However, in integrating blueberries and cranberries into commercial formulations, manufacturers must carefully consider trade-offs between enhancing nutritional content and maintaining desired sensory properties. This is important to balance the shelf life of the product, its production costs, and market demand. Moreover, it is very beneficial to educate consumers about the benefits of fortified muffins. Improving marketing strategies to emphasize the improved taste and potential health benefits of these muffins can enhance their appeal to health-conscious consumers.

## 4. Conclusions

The addition of blueberry and cranberry powders has influenced the physicochemical characteristics of muffins, especially specific gravity, moisture, pH, and acidity. Significantly, the addition of cranberries and blueberries was observed to decrease the values of lightness and yellowness and to increase the values of redness. The higher content of these powders has caused higher values of hardness and gumminess, while an increase in the values of polyphenols has also been observed, but not in a significant way. Higher polyphenol values contribute to the antioxidant properties of muffins when blueberry and cranberry powders are incorporated into their formulation. These antioxidants help extend the muffins' shelf life by combating oxidative damage and enhance dough texture, resulting in a better overall product. Incorporating these berries into muffin formulations not only enhances their antioxidant properties but also introduces potential health benefits, which may include improved cardiovascular health, reduced inflammation, and enhanced antioxidant defense mechanisms. In addition to these analyses, cranberry- and blueberry-enriched muffins have been evaluated by sensory evaluators who have shown a greater liking for muffins with a higher concentration of added extracts. In particular, samples with 9% cranberry and 6% blueberry were distinguished as the most liked, likely due to their vibrant and intense color, which was particularly attractive to consumers in the evaluation. Conversely, the control samples and those with 3% extracts were the least liked, mainly because the taste and appearance did not appeal as much to the participants. These results show that cranberries and blueberries are a good option to increase the nutritional value and general acceptance of muffins as bakery products enjoyed by different age groups. The findings of this study would help promote the use of different plant-based by-products rich in various bioactive compounds, such as food ingredients. However, further research could explore the incorporation of other types of berry powders, such as raspberry, strawberry, or blackberry, to assess their impact on muffin properties and nutritional profile. Additionally, long-term stability studies investigating the retention of added nutrients during the shelf life of the muffins would be valuable for assessing their potential health benefits over time. Furthermore, conducting consumer perception studies on a larger scale or across different demographic segments could provide deeper insights into market acceptability and preferences for enriched muffins with blueberry and cranberry powders.

## Figures and Tables

**Table 1:** Composition of control and enriched muffins.

**Ingredients**	**CM**	**M1**	**M2**	**M3**
White flour (g)	100	97	94	91
Yogurt (mL)	133.33	133.33	133.33	133.33
Eggs (mL)	17.67	17.67	17.67	17.67
Sunflower oil (mL)	33.33	33.33	33.33	33.33
White sugar (g)	66.67	66.67	66.67	66.67
Vanilla extract (g)	3.33	3.33	3.33	3.33
Baking powder (g)	4	4	4	4
Blueberry/cranberry powder (g)	—	3	6	9

Abbreviations: CM = muffins without blueberry/cranberry powder, g = gram, M1 = 3% powder muffins, M2 = 6% powder muffins, M3 = 9% powder muffins, mL = milliliters.

**Table 2:** Nutritional information per 100 g of powdered blueberry and cranberry.

Blueberry powder	Carbohydrates	60.4 g
	Fiber	19.5 g
	Fat content	6.5 g
	Protein	4.7 g
	Salt	30.2 mg
	Vitamin K	53.3 mg
	Vitamin C	42.6 mg
	Vitamin E	11.2 mg

Cranberry powder	Carbohydrates	2 g
	Fiber	6 g
	Fat content	0.2 g
	Potassium	28.23 mg
	Calcium	4.46 mg
	Manganese	1.56 mg
	Vitamin C	5.71 mg
	Vitamin E	0.23 mg

**Table 3:** Physicochemical parameters of muffins enriched with blueberry and cranberry powder.

**Sample**	**P (%)**	**Weight (g)**	**Height (cm)**	**Specific gravity (g/cm ** ^**3**^ **)**	**Moisture (%)**	**Water activity**	**pH**	**Acidity**
Control		51.99 ± 1.14^a^	3.20 ± 0.52^a^	0.88 ± 0.01^b^	21.58 ± 1.83^b^	0.73 ± 0.06^a^	5.82 ± 0.03^a^	1.80 ± 0.00^c^

Muffins enriched with blueberry	3%	52.40 ± 5.29^a^	3.80 ± 0.10^a^	0.86 ± 0.05^b^	24.04 ± 1.85^ab^	0.77 ± 0.01^a^	5.54 ± 0.03^b^	2.27 ± 0.12^b^
	6%	55.53 ± 1.50^a^	3.53 ± 0.15^a^	0.92 ± 0.02^ab^	25.03 ± 0.43^a^	0.78 ± 0.01^a^	5.39 ± 0.02^c^	2.33 ± 0.12^ab^
	9%	56.97 ± 1.42^a^	3.63 ± 0.32^a^	0.94 ± 0.04^a^	24.01 ± 0.61^ab^	0.77 ± 0.02^a^	5.38 ± 0.02^c^	2.47 ± 0.12^a^

Muffins enriched with cranberry	3%	74.42 ± 2.50^a^	5.13 ± 0.23^a^	0.98 ± 0.01^a^	28.95 ± 1.84^a^	0.71 ± 0.01^a^	5.79 ± 0.03^b^	2.27 ± 0.23^bc^
	6%	72.85 ± 3.68^a^	4.60 ± 0.10^b^	1.01 ± 0.02^a^	30.52 ± 1.27^a^	0.72 ± 0.02^a^	5.41 ± 0.05^c^	2.80 ± 0.20^ab^
	9%	73.44 ± 2.75^a^	4.73 ± 0.06^b^	0.97 ± 0.02^a^	30.51 ± 1.14^a^	0.72 ± 0.01^a^	5.26 ± 0.02^d^	3.00 ± 0.20^a^

*Note:* Data are expressed as mean ± standard deviation. Means labeled with different superscripts within the same column indicate significant differences (*p* < 0.05).

Abbreviation: P = powder.

**Table 4:** Color attributes of muffin crust enriched with blueberry and cranberry powders.

**Attribute**		**Type of plant extract**		**p** ** value**
		**Cranberry**	**Blueberry**	
Lightness (*L*^∗^)	Control	70.83c	70.83d	
	3%	68.60bB	60.48cA	< 0.001
	6%	64.93aB	55.09bA	< 0.001
	9%	65.37aB	51.63aA	< 0.001

Redness (*a*^∗^)	Control	−0.13a	−0.13a	
	3%	2.12bA	4.48bB	< 0.001
	6%	3.54cA	7.11cB	< 0.001
	9%	3.82cA	7.58cB	< 0.001

Yellowness (*b*^∗^)	Control	26.88c	26.88c	
	3%	22.85bB	14.01bA	< 0.001
	6%	13.05aB	8.43aA	< 0.001
	9%	14.68aB	7.27aA	< 0.001

Chroma (*C*^∗^)	Control	26.90c	26.90c	
	3%	22.95bB	14.71bA	< 0.001
	6%	13.52aB	11.10aA	< 0.01
	9%	15.20aB	10.59aA	< 0.001

*h* value (*h*)	Control	90.88c	90.88c	
	3%	84.73bB	71.98bA	< 0.001
	6%	74.80aA	48.91aA	< 0.001
	9%	74.99aA	43.05aA	< 0.001

*Note:* Different lowercase letters in the same column denote significant differences in the percentage of blueberry and cranberry powders (*p* < 0.05). Different uppercase letters in the same row indicate significant differences for the type of plant extract (*p* < 0.01, *p* < 0.001).

**Table 5:** Color attributes of muffin crumbs enriched with blueberry and cranberry powders.

**Attribute**		**Type of plant extract**		**p** ** value**
		**Cranberry**	**Blueberry**	
Lightness (*L*^∗^)	Control	72.29c	72.29d	
	3%	62.05bB	55.24cA	< 0.001
	6%	58.25aB	50.63bA	< 0.001
	9%	57.13aB	46.83aA	< 0.001

Redness (*a*^∗^)	Control	−3.60a	−3.60a	
	3%	0.94bA	4.75bB	< 0.001
	6%	3.64dA	7.25cB	< 0.001
	9%	2.94cA	7.69cB	< 0.001

Yellowness (*b*^∗^)	Control	18.08d	18.08d	
	3%	14.26cB	6.83cA	< 0.001
	6%	10.69aB	3.80bA	< 0.001
	9%	12.19bB	2.53aA	< 0.001

Chroma (*C*^∗^)	Control	18.43d	18.43b	
	3%	14.30cB	8.34aA	< 0.001
	6%	11.31aB	8.21aA	< 0.001
	9%	12.54bB	8.11aA	< 0.001

*h* value (*h*)	Control	101.26d	101.26d	
	3%	86.21cB	55.09cA	< 0.001
	6%	71.07aB	27.79bA	< 0.001
	9%	76.34bB	18.98aA	< 0.001

*Note:* Different lowercase letters in the same column denote significant differences in the percentage of blueberry and cranberry powders (*p* < 0.05). Different uppercase letters in the same row indicate significant differences for the type of plant extract (*p* < 0.01, *p* < 0.001).

**Table 6:** Total color difference (*E*) between muffins enriched with blueberry and cranberry powder.

	**Treatment**	**Control**	**PE 3%**	**PE 6%**	**PE 9%**
Crust of cranberry muffins	Control	—	V	V	V
	BP 3%	5.13	—	V	V
	BP 6%	15.48	10.56	—	II
	BP 9%	13.94	8.95	1.71	—

Crumb of cranberry muffins	Control	—	V	V	V
	BP 3%	11.83	—	V	V
	BP 6%	17.44	6.06	—	II
	BP 9%	17.53	5.70	1.99	—

Crust of blueberry muffins	Control	—	V	V	V
	BP 3%	17.15	—	V	V
	BP 6%	25.31	8.19	—	IV
	BP 9%	28.51	11.55	3.68	—

Crumb of blueberry muffins	Control	—	V	V	V
	BP 3%	22.07	—	V	V
	BP 6%	28.12	6.06	—	IV
	BP 9%	31.89	9.89	4.03	—

*Note:* I, II, III, IV, and V signify the categories of total color difference (*E*). Category I: 0 < Δ*E* < 1, indicating that there is no perceivable difference. Category II: 1 < Δ*E* < 2, where only experienced observers can detect a difference. Category III: 2 < Δ*E* < 3.5, noticeable even to inexperienced observers. Category IV: 3.5 < Δ*E* < 5, representing a clear color difference. Category V: Δ*E* > 5, where two distinct colors are observed.

Abbreviation: BP = berry powder.

**Table 7:** Texture attributes of muffins enriched with blueberry and cranberry powders.

**Attribute**	**Percentage**	**Type of plant extract**		**p** ** value**
		**Cranberry**	**Blueberry**	
Hardness	Control	8.79a	8.79a	
	3%	14.83bB	10.12aA	< 0.001
	6%	14.27bB	10.47aA	< 0.001
	9%	15.32bB	10.58aA	0.001

Cohesiveness	Control	0.343b	0.343a	
	3%	0.334bA	0.336aA	0.898
	6%	0.307abA	0.336aB	0.01
	9%	0.285aA	0.318aB	< 0.05

Gumminess	Control	3.02a	3.02a	
	3%	4.94bB	3.42aA	< 0.001
	6%	4.39bB	3.49aA	< 0.01
	9%	4.39bB	3.38aA	< 0.05

*Note:* Different lowercase letters in the same column denote significant differences in the percentage of blueberry and cranberry powders (*p* < 0.05). Different uppercase letters in the same row indicate significant differences for the type of plant extract type (*p* > 0.05, *p* < 0.05, *p* < 0.01, and *p* < 0.001).

**Table 8:** Content of total phenolic compounds (TPC).

	**Type of plant extract**	
	**Cranberry**	**Blueberry**
Powder	1907.04^a^	2394.81^a^
Control	828.89^b^	828.89^b^
3%	877.78^b^	1055.56^b^
6%	942.59^b^	1082.22^b^
9%	928.89^b^	1695.56^ab^

*Note:* Different superscript letters denote significant differences in the percentage of blueberry and cranberry powders (*p* < 0.05).

## Data Availability

All data are provided in this manuscript.
